# Effect of *Cichorium intybus* on production performance, carcass quality and blood lipid profile of hybrid duck

**DOI:** 10.5713/ab.22.0041

**Published:** 2022-09-02

**Authors:** Nafiatul Umami, Eka Rizky Vury Rahayu, Bambang Suhartanto, Ali Agus, Edi Suryanto, Mohammad Mijanur Rahman

**Affiliations:** 1 Faculty of Animal Science, Universitas Gadjah Mada, Yogyakarta, 55281, Indonesia; 2 Faculty of Agro Based Industry, Universiti Malaysia Kelatan, 17600 Jeli, Kelatan, Malaysia

**Keywords:** Carcass Quality, *Cichorium intybus*, Histomorphology, Hybrid Ducks, Lipid Profile, Performance

## Abstract

**Objective:**

One hundred hybrid male ducks (Mojosari×Alabio) were used to examine the efficacy of chicory supplementation as nutritional feed manipulation on production performance, and blood lipid profile of hybrid ducks.

**Methods:**

The ducks were tagged, weighed, and then allotted randomly to one of the four treatment diets using a completely randomized design. The experimental diets were: i) P0 (100% basal diets+0% chicory as control), ii) P1 (95% basal diets+5% chicory), iii) P2 (90% basal diets+10% chicory) and iv) P3 (85% basal diets+15% chicory). For each treatment group, there were 5 replicates of 5 birds each. All experimental diets were isonitrogenous and isocaloric using locally available ingredients.

**Results:**

Hybrid ducks with fed diets supplemented fresh chicory (5%, 10%, and 15%) showed increased feed intake and body weight gain, as well as feed conversion ratio to be smaller than those ducks fed diets without chicory supplementation (control). The ducks fed 10% chicory supplementation contained significantly (p<0.05) lower ash and higher organic matter contents of meat than those ducks fed other diets. The ducks fed 15% chicory supplementation showed the lowest crude protein and cholesterol content of meat among the treatment diets. Ducks fed chicory supplementation showed lower (p<0.05) blood cholesterol and triglyceride levels than those ducks fed without chicory supplementation, while dietary interventions had no major (p>0.05) influence on low-density lipoprotein and high-density lipoprotein levels in duck blood.

**Conclusion:**

In this study, 10% chicory supplementation showed the best results characterized by an increase in growth performance, carcass quality, small intestinal histomorphology, and lower cholesterol levels of meat.

## INTRODUCTION

Indonesia is a tropical country that has a high diversity of biological resources, as well as its poultry genetic resources. The large number of poultry genetic resources in Indonesia is not accompanied by utilization through cultivation to meet the need for protein food in the community. In Indonesia, ruminants such as cattle, goats, and sheep, as well as poultry such as chicken, Muscovy duck, and duck, are the most common sources of meat. Duck is an excellent source of animal protein. Compared to other poultry, ducks are more resistant to disease. They have a low risk of death and have meat with a savorier taste than chicken.

Duck is a species of poultry that produces eggs and meat. This type of poultry produces meat with a different carcass composition from other poultry such as chicken and turkey. One of the duck's characteristics as waterfowl is the formation of higher subcutaneous fat compared to other poultry [1]. This is a form of adaptation to maintain their body while swimming. Chicken meat has a lipid content of 15% of body weight, whereas duck has 25% to 30%, and turkey has 8% to 15% meat lipid depending on age and gender [2]. Meat that contains a high lipid can cause a high risk of disease [3]. Public awareness about human health increases the demand for poultry meat that is safe for consumption. Strategies to reduce lipid content in duck carcasses and optimize their growth can be done through nutrition feed manipulation. The study of Ouyang et al [4] through feed manipulation with supplementation alfalfa flavonoids at 5, 10, 15 mg/kg showed decreased the total cholesterol and low-density lipoprotein (LDL) levels and increased high-density lipoprotein (HDL). Another study, supplementation 3% fresh alfalfa in the diet showed an increase feed consumption and body weight gain, but supplemented at 6% did not increase live weight, carcass weight, and carcass percentage of hybrid duck [5,6]. The application of forage as a feed supplement in duck not only will benefit gastrointestinal development, but also will economize traditional ingredients which are becoming costly or less available.

Chicory plant (*Cichorium intybus*) is a feed that is easy to cultivate both monoculture and intercropping [7]. Chicory can be harvested more than once in one planting. Chicory is known as an herbal plant that has potential as animal feed besides having high quality and drought resistance. Chicory plant contains phytochemicals that are beneficial for livestock health. Insulin, volatile components (monoterpenes and sesquiterpenes), esculin, flavonoids, coumarins, and vitamins are all found in the chicory plant [8]. The polyphenol content in chicory plants expresses various activities that can improve health such as antiviral, anticarcinogenic, antibacterial, anti-inflammatory, antifungal, antimutagenic, immunostimulant and antioxidant. Furthermore, it can maintain the gastrointestinal tract and help to lower blood cholesterol levels [9]. Flavonoids in chicory plants can boost poultry productivity when added to the diet. Isoflavone content can enhance average body weight gain (BWG)[10].

These flavonoids trigger a combination of growth hormone and hepatic growth hormone receptors, which leads to an increase in growth. It leads insulin concentrations to increase, and then improves livestock growth. Chicory plants are fibrous plants but have the potential to be used as feed for ducks because ducks harbor fiber-degrading microorganisms in their digestive tract. Chicory in duck diet triggers peristaltic motions in the digestive system. This, in turn, will improve nutrient absorption in the small intestine, enhancing animal productivity [11].

There is a lack of studies on the comparison of chicory supplementation in the form of basal feed. Therefore, this research was carried out to determine the effect of chicory on hybrid ducks’ performance, blood lipid profile, and carcass quality.

## MATERIALS AND METHODS

### Forage preparation

The forage used in this study was *Cichorium intybus* var. Chico. The seeds of chicory were sown in 13 plots, with different planting times for each plot. The area of each plot was 3 m×2 m with distance between plots of 0.5 m. The seeds were sown by scattering at a seedling rate of 0.1 g/m^2^ or 60 seeds/m^2^. The seed that had been sown were then covered with soil. The sowing was executed by putting 7 to 10 cm between the edges of the plots. Chicory was maintained until 30 days, and cut with rotational defoliation method to maintain it in a fresh condition. Chicory was cut at 4 cm above the ground level. Furthermore, fresh chicory leaf was fed finely chopped. So, the production of each plot has been estimated to be sufficient for experimental dietary treatment for four days.

### Animal care

The research was conducted at the Faculty of Animal Science, Universitas Gadjah Mada from April to July 2021. The ethical feasibility commission approved this research protocol for preclinical testing with licensing number 00074/EC-FKH/Eks./2021 from the Faculty of Veterinary Medicine, Universitas Gadjah Mada, Yogyakarta, Indonesia.

### Experimental livestock

One hundred hybrid male ducks (Mojosari×Alabio) were used in this study. All ducks were tagged, weighed, and then allotted randomly to one of the four treatment dietary groups using a completely randomized design. For each treatment, there were 5 replications of 5 birds each. This study made use of 20 battery cages. Each cage was measured (1.4 m×1.2 m×0.5 m) and equipped with a feeder and drinker (PT. Medion Ardhika Bhakti, Bandung, Indonesia). The rearing phase started when the ducks were 1 week old with an initial weight of 150 grams and then kept for 50 days. Eye drops vaccine type ND-B1 was given ducks at 5 days old, and ND Lasota was given ducks through drinking water at 10 days old. Forty ducks at 57 days old were randomly selected (two per replication) for slaughtering and sampling. Blood samples were taken before the ducks were slaughtered.

### Experimental diets

The diet consisted of corn, bran, soybean meal, fish meal, pollard, and premix. Chemical composition of the feed ingredients is shown in Table 1. Furthermore, the use of chicory in this study was based on the crude fiber (CF) digestibility tolerance level of ducks [10]. The feed ingredients were mixed and finely chopped fresh chicory leaf added to the four experimental dietary treatments: i) P0 (100% basal diets+0% chicory as control), ii) P1 (95% basal diets+5% chicory), iii) P2 (90% basal diets+10% chicory), and iv) P3 (85% basal diets +15% chicory). Each dietary treatment was prepared as iso-nitrogenous and iso-caloric. The experimental diets were prepared based on the broiler duck's nutritional requirements as determined by the NRC [12] including 2,900 MJ/kg energy, 22% crude protein (CP), 0.65% calcium, 0.9% lysine, 0.4% methionine and 0.23% tryptophan. The feed was given 2 times a day, namely every morning and evening at 07:00 and 16:00. Feed and drinking water were provided *ad libitum*. Feed formulation based on nutrient composition is shown in Table 2.

### Growth performance and carcass quality

Performance of ducks was measured throughout the study including feed intake, BWG, and feed conversion ration (FCR). Feed intake was calculated by subtracting the amount of feed given from the leftover feed each day (g/head) [5]. The body weight (BW) of ducks was recorded at the start, 1 week interval and at the end of the experiment (g/head). The FCR was determined by dividing feed intake by BWG [10]. Carcass quality parameters were measured on the last day of the experiment (50 day). Before slaughter, the ducks were stopped feeding Fed was withheld for 12 hours but water was available *ad libitum*, and then the ducks were weighed to determine the BW [1] prior to slaughter. The method of slaughtering ducks was performed according to SNI 99002:2016 [13] with the halal method without stunning. The blood was removed completely, and then weighed to determine the weight after slaughter. Similarly, the hair removal, offal removal, without head, neck, legs, lungs, and/or kidneys for carcass evaluation was performed according to SNI 3924:2009 [14]. Carcass percentage was obtained by multiplying the ratio of carcass weight to BW by 100%.

### Gastrointestinal measurement

The digestive organs were removed from the slaughtered duck. Gastrointestinal measurements were taken including the length, weight, and pH of the digestive tract. Immediately, each part of the digestive tract was measured using tape measure (cm), followed by weighing the organs that had been cleaned from digesta. For the measurement of pH, organs were cut a little and measured with a pH meter. The sections measured were included the gizzard, duodenum, jejunum, ileum, cecum, colon, pancreas, and liver.

### Meat quality

The meat pH was obtained after the meat was chopped (2 g) and measured using a calibrated pH meter digital (PL-600; pH/mV/Temp Meter, Taipe, Taiwan). The amount of water bound by the meat was calculated using the Hamm method to determine water holding capacity (WHC, %) [15]. Cooking loss (%) was calculated by subtracting the mass of the meat after heating/cooking. Meat tenderness (g/cm^2^) was measured using Breakdown Warner-Blatzler (WB). The meat chemical quality (%) was measured following the method of AOAC [16], including dry matter (DM), ash, organic matter (OM), CP, CF, and extrach ether (EE). Cholesterol levels of breast, thigh, and liver meat were taken on the right side of the duck. Cholesterol levels (mg/100 g) was analyzed based on the Lieberman Burchard method [17].

### Intestinal morphometry

Forty fresh jejunal intestine samples that had been cut 2 cm at the middle part were cleaned, fixed with 10% buffer-formalin, soaked for 24 to 48 hours, and then made as histological specimens. Samples were hydrated using graded alcohol (70%, 80%, 90%, and 100%) and left to soak for 10 seconds. The sample was then put in xylol and dipped in paraffin. The sample was thinly sliced using a microtome with a thickness of 5mm and painting was done by the hematoxylin-eosin method (HE). Finally, the sample was observed under a microscope. Villus height was measured from the tip to the villus crypt junction (μm). Villus width was measured apical and basal width (μm). The crypt depth was measured as the depth between adjacent villi (μm). Intestinal morphometry were determined as reported by Sadeghi et al [18]. With Optilab Viewer 2.2 (PT. Miconos Transdata Nusantara, Yogyakarta, Indonesia) linked to a laptop display, sections were examined at 4× magnification.

### Blood lipid profile

Blood lipid profiles including cholesterol, triglyceride, LDL, and HDL levels were estimated by photometric technology. CHOD PAP method (mode of reaction: end point; linearity: 600 mg/dL) was used to estimate total cholesterol. Enzymatic calorimetric method GPO PAP was used to estimate triglycerides. Enzyme selective solubilization method (mode of reaction: end point; linearity: 150 mg/dL) was used to estimate HDL. Homogenous enzymatic colorimetric assay with rapid reagent kit (mode of reaction: end point; linearity: 700 mg/dL) was used to estimate LDL. Calibrators received with the testing kits were used for the assay. Stringent internal quality control checks were performed regularly [19].

### Data analysis

Statistical analysis used R software version 4.1.1 with a 5% level of significance. A descriptive test was applied to identify the characteristics of data observation comprising of minimum, maximum, mean, and standard deviation. The Shapiro-Wilk test was used to assess data normality, whereas the Bartlet's test was used to assess variance homogeneity. Comparison mean of observed parameters among treatments was tested using analysis of variance and followed by Duncan Multiple Range Test. The analysis of multicriteria using the ranking and rating method was also used to determine the most optimum dose of chicory as supplemental materials for duck feed. The use of multicriteria analysis was directed to consider many indicators that become the main considerations for evaluating the duck response to chicory treatments. In this context, eight indicators had to be considered to determine the best chicory treatment and every indicator had different interpretations in management practice.

## RESULTS AND DISCUSSION

### Growth performance and carcass quality

Growth performance is an essential criterion for evaluating if a treatment has a positive or negative effect on the experimental animals. In this study, chicory supplementation influenced growth performance and carcass quality of hybrid ducks considerably (p<0.05) (Table 3).

Hybrid duck with fed diets supplemented fresh chicory (5%, 10%, and 15%) showed increased feed intake and body weight gain, as well as feed conversion ratio to be smaller than those ducks fed diets without chicory supplementation (control). This is possibly related to the CF level of hybrid ducks’ digestible diets. This means that 10% chicory supplementation can still be tolerated by ducks, which is indicated by an increase in carcass performance and quality. According to Suwignyo et al [10], ducks have a higher tolerance for CF than other poultry. In this study, 10% chicory supplementation showed the best results on growth performance, but 15% chicory supplementation decreased its growth performance. The CF concentration which tends to be high in the feed causes nutrient digestion to take additional time in the digestive organs, consequently lowering its energy value. Feeds with high CF are bulky which can cause ducks to feel full, thereby reducing feed consumption. Under normal physiological conditions, the amount of feed consumed will always be followed by an increase in BW. Potential response to CF may be affected by the source and level of fiber supplementation in the feed, the nature of the feed, the physiological status, and animal health. The CF content of the feed can cause differences in the rate of feed in the digestive organs, the pH value, and the production of volatile fatty acids in poultry [20]. The BWG is a major factor influencing FCR, with low BWG leading to high FCR and conversely. The FCR illustrates the production efficiency, where smaller FCR value shows an effective application [10]. In this study, 10% chicory supplementation showed better FCR compared to other treatments. This was because of the low feed intake; ducks were able to produce high BW.

Carcass quality which included BW, weight after slaughter without blood, carcass weight, and carcass percentage is easiest or simplest measured by weighing the ducks individually. Body weight is an economic parameter in livestock cultivation and is the result of accumulated growth during maintenance which is influenced by feed. In this study, the carcass quality increased according to higher chicory levels. However, at a certain level, there was variation in carcass quality produced. This was due to nutrient feed that can be digested in the digestive tract, one of which was CF. This means that 10% chicory supplementation was still tolerable by ducks, which was shown by a rise in carcass performance and quality. In this study, chicory supplementation increased the BW of hybrid ducks. This was because BW is related to BWG. The BWG is affected by the nutrient content of the ration given, the consumption of the ration, the livestock health condition, the environmental temperature, and sex [9]. The BW is also closely related to livestock weight after slaughter. Higher carcass weight in this study had a close relationship with BW, as the BW increased, carcass production tended to increase. Carcass percentage is a parameter to measure livestock production and is related to BW and BWG. These outcomes corresponded to Gariglio et al [21] findings where the carcass percentage is directly proportional to BW, where increasing BW tends to produce a high carcass percentage as well. The carcass percentage will increase on older ducks and higher BW, as the body proportion other than the carcass was decreased. The body proportion other than the carcass decreases because that section contains more bones.

### Gastrointestinal measurement

**Figure 1:**
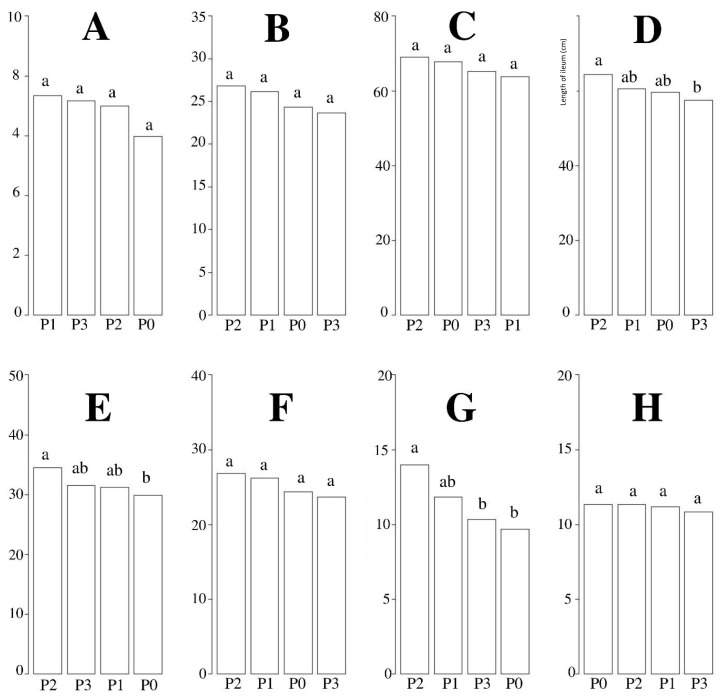
Measurement of length (cm) digestive organs after chicory supplementation. (A) gizzard; (B) duodenum; (C) jejunum; (D) ileum; (E) cecum; (F) colon; (G) pancreas; (H) liver. P0, 100% basal diets+0% chicory; P1, 95% basal diets+5% chicory; P2, 90% basal diets+10% chicory; P3, 85% basal diets+15% chicory. ^a,b^ Means with different superscripts in a row differ significantly (p<0.05).

Figure 1 presents the results of hybrid ducks with chicory supplementation on the length of the digestive tract. The results indicated that chicory supplementation had significant (p<0.05) effects on the length of the ileum, cecum, and pancreas of hybrid ducks. The 10% chicory supplementation showed the best results among all other treatments.

The small intestine is where digestion and absorption of digestive products occur. Various enzymatic processes take place in the small intestine that contribute to expedite and streamline the breakdown of carbs, proteins, and lipids to improve absorption. If feed consumption increases, the length and surface area of the intestine will expand because the intestine has increased the nutrients absorption process. Villi contained in the intestine play an important role in the nutrient absorption process. The duodenum's functions are to digest and break down nutrient levels in the form of starch, lipid, and protein. The nutrients in the duodenum have not occurred optimally because there are still macronutrients that cannot be absorbed by the intestinal villi. Absorption of glucose, fatty acids, and amino acids occurs maximally in the jejunum [22]. The ileum is the last part of the small intestine and serves mostly as a place for water and mineral absorption, though some nutrient absorption occurs here as well. Increased intestinal length improves nutrient absorption.

Table 4 presents data for measuring organ weight and digestive tract pH of hybrid ducks with chicory supplementation. The results indicated that chicory supplementation significantly (p<0.05) affected the weight (gizzard and cecum) and pH (gizzard and colon) of hybrid ducks. The 15% chicory supplementation showed the best results among all other treatments. Duck growth and performance are typically affected by the growth and function of the digestive tract. The increasing of chicory in the diets will be increase the intake of CF. This can impact on the performance of the organs (gizzard and cecum) which will be heavier due to digesting CF, so the organs are growing. A bigger gizzard can improve digestion and intestinal digestion efficiency [23]. Under proper feed flow regulation, gizzard growth can enhance small intestine function [22]. Chicory supplementation significantly (p<0.05) affected the pH of gizzard and colon hybrid ducks. Duodenum and jejunum had an acidic pH ranging between 4 to 5 and 5 to 6, the ileum had a near-neutral pH ranging from 6 to 7. The degree of acidity (pH) will reduce the growth of pathogenic bacteria which causes the rate of feed, nutrient digestibility, and nutrient absorption processes to run optimally [24]. The large intestine is a segment of the digestive tract that functions to reabsorb nutrients that have not been absorbed in the previous segment of the digestive tract and to channel the remaining digestive products into the cloaca. Digestion that occurs in the large intestine is due to enzymes from the small intestine and enzymes produced by microorganisms found in the large intestine. The cecum is a segment of the digestive tract in which the absorption of a small amount of water, carbohydrates, and protein is assisted by bacteria. Cecum functions to digest CF assisted by cellulolytic bacteria which produce short-chain fatty acid [24].

### Average physical, chemical, and cholesterol quality of hybrid duck meat

Meat quality is an important factor that influences the selling price and consumer preferences. The physical, chemical, and cholesterol qualities of hybrid duck meat after chicory supplementation are shown in Table 5. Chicory supplementation showed significant (p<0.05) effects on WHC, moisture content, DM, OM, EE, and CP in hybrid ducks. The 10% chicory supplementation had a noticeably (p<0.05) effects on the ash and OM contents of duck meat. The 15% chicory supplementation showed the lowest CP and cholesterol content of duck meat among all other treatments.

Chicory supplementation in this study increased (p<0.05) the WHC of hybrid duck meat. The WHC increased at the level of 5% and 10% chicory supplementation, but at the level of 15%, it tended to decrease. This means that 10% chicory supplementation can be tolerated by hybrid ducks characterized by increasing WHC content. The pH of the meat produced might generate high WHC levels. Protein denaturation can occur when the pH drops. Protein denaturation leads a drop in protein solubility, which causes a decline in WHC. High WHC produces good quality meat. The WHC in this study was within normal limits of 20% to 60%. In this study, chicory supplementation did not affect (p>0.05) hybrid duck meat cooking loss. Cooking loss is influenced by pH value, length of muscle fiber sarcomere, length of the fiber cut, myofibril contraction status, sample size and weight, cross-section of meat, heating, variations related to meat fat, age, and energy consumption in feed. The cooking loss value is directly associated to the water holding capacity. High WHC will result in lower cooking losses. Meat tenderness is strongly associated with meat boiling or cooking process [25]. Chicory supplementation did not affect the meat tenderness because there was no change in the protein structure of the meat. Boiling meat damages and alters the structure of muscle proteins, particularly actin and myosin. The meat changing process is one way to soften meat by cooking which can cause protein denaturation. Protein denaturation is the process by which proteins are broken down into smaller parts. Meat tenderness is affected by the postmortem factor by cooking by boiling. Chicory supplementation in this study had no significant (p>0.05) effect on the pH of hybrid duck meat. This is because the feed given is iso carbohydrate in all treatments. The use of high carbohydrates feed can affect glycogen levels in the muscles so that it affects the pH of the meat. Higher chicory supplementation can lower the pH of the meat [26].

Chicory supplementation in this study increased the moisture content of the hybrid duck meat. Water is an extracellular constituent and a critical element over all animal tissues. Water is a universal medium, and broiler meat moisture content is about 65% to 80%. Long-term storage of duck meat results in higher in water content. It is due to the release of bound water into free water with increased microbial activity. Chicory supplementation reduced (p<0.05) the DM content of hybrid duck meat, but at 5%, 10%, and 15% levels of chicory supplementation did not show the same results (p>0.05). This was due to various types of feed given between chicory and control supplemented feed. Chicory (0%) was not added to the control feed, indicating that the DM of the feed tended to be higher. The composition of the feed consumed by livestock will affect the quality of the meat produced and vice versa. Chicory contains saponins, flavonoids, and tannins that have the potential to reduce lipid accumulation, so in this study, hybrid ducks with chicory supplementation showed lower meat lipid content than control [8]. Crude lipid content of broiler chicken breast was 1.81% to 2.31%. Chicory supplementation of 15% showed lower CP than other treatments. This was because the protein bound to the cell wall is processed in the digestive tract and requires microbes to degrade fiber for a long time. Protein-bound in the cell wall is excreted through the feces.

In this study, 15% chicory supplementation showed the lowest (p<0.05) chest cholesterol among all other treatments. The low meat cholesterol was caused by the saponin content in chicory. Saponins can impede the incorporation of cholesterol into micelles and hence its absorption in the small intestine. Cholesterol levels can be influenced by the percentage of abdominal fat, ration consumption, and low protein consumption so that maximum growth is not achieved and causes low cholesterol formed in the body. If the lipid content in the body is high, the cholesterol is high. The CF content in the ration can also reduce cholesterol levels in ducks. Lipid content is positively correlated with cholesterol, so a higher lipid content in poultry feed will produce a higher meat cholesterol content and vice versa. Cholesterol is part of a lipid, when the lipid content in the body increases, therefore the cholesterol level increases [27]. Saponins are natural detergents or glycosides. The foaming of saponins is caused by the incorporation of hydrophobic sapogenins and hydrophilic sugar chains [28]. The hypocholesterolemic effect of saponins has a counter effect on the absorption of cholesterol and bile salt derivatives. The planar steroid ring core of saponins interacts with the planar ring of cholesterol and bile salts, thereby blocking the absorption. According to Ferreira et al [28] there are several ways by which saponins or tannins lower cholesterol. One of them is by inhibiting cholesterol absorption by raising the cholesterol excretion through feces. Saponins bind bile salts in the gastrointestinal tract that cause non-absorption of salt bile and increase the excretion of fecal bile salts and accelerate the synthesis of endogenous bile salts which ultimately lowers cholesterol levels.

### Small intestinal morphometric characteristic

**Figure 2:**
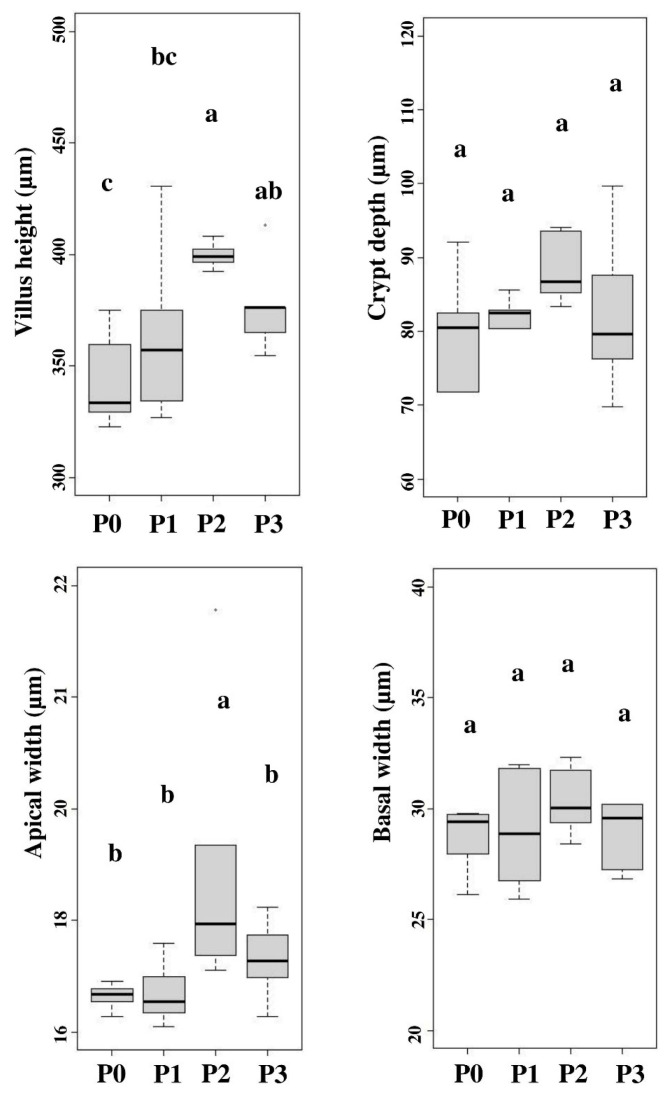
The histopathology of the small intestine included villus height, apical width, basal width and crypt depth. P0, 100% basal diets+0% chicory; P1, 95% basal diets+5% chicory; P2, 90% basal diets+10% chicory; P3, 85% basal diets+15% chicory. ^a–c^ Means with different superscripts in a row differ significantly (p<0.05).

Chicory supplementation significantly (p<0.05) influenced the height of the jejunum villi in hybrid ducks. The villi in the 10% chicory supplementation group showed higher apical width than other treatments, while basal width and crypt depth in all treatments showed the same results (p>0.05). Figure 2 shows the mean gut histomorphology (villi height, apical width, basal width, and crypt depth) of hybrid ducks after chicory supplementation. Intestinal villi play an important function in nutrition absorption in the gut. The small intestine surface area such as the height of the villi describes the area for absorption of nutrients. The morphological structure of the intestine is one of the indicators used to evaluate growth quality [27]. Higher small intestinal villi height provides a large surface area between erythrocytes and nutrients, resulting a better nutrient absorption [20]. The increase in the height and width of the villi increased the efficiency of nutrient transport in the hybrid duck body. The digestive tract flow rate affects the increase in the villi width. Intestinal activity can influence the enlargement in the villi surface width to absorb nutrients. Intestinal villi in poultry can grow optimally if the nutrient requirements during their growth period are met. Nutrient absorption in the intestine can trigger the dilation of the villi. The development in intestinal villi height suggests that the small intestine is digesting nutrition efficiently. Efficient intestinal absorption represents a healthy gut function, which can improve nutrient absorption [29].

Chicory supplementation increased feed CF levels. Crude fiber content can affect intestinal villi development [5]. Insufficient CF concentration may be a contributing factor to the underdevelopment of intestinal villi height and width. As a result, nutrient absorption is not optimal. The CF supplementation in feed can aid the feed digestion and villi expansion. Dietary fiber concentration affects the alteration in animal intestinal morphology, mainly villi surface (width and height) and number [5]. Increased villi surface area associated with elevated mucosal cell proliferation rate [30]. Table 5 shows that fresh chicory supplementation resulted in higher consumption than the control treatment. Jiang et al [3] stated that feed consumption affects the height of the small intestinal villi and into the crypts. Increased feed consumption stimulates the small intestine development, which may be because of the influence of some fibrous components on the of microorganism composition in the digestive tract, particularly the small intestine and cecum, via fermentation, thus further enhancing the villi height and crypt depth in poultry small intestine. Fiber addition in feed promotes the synthesis of volatile fatty acids, a result of microbial fermentation in the small intestine and cecum. On the other hand, butyric acid can increase the production of small intestinal cells, which can increase crypts depth.

### Blood lipid profile

**Figure 3:**
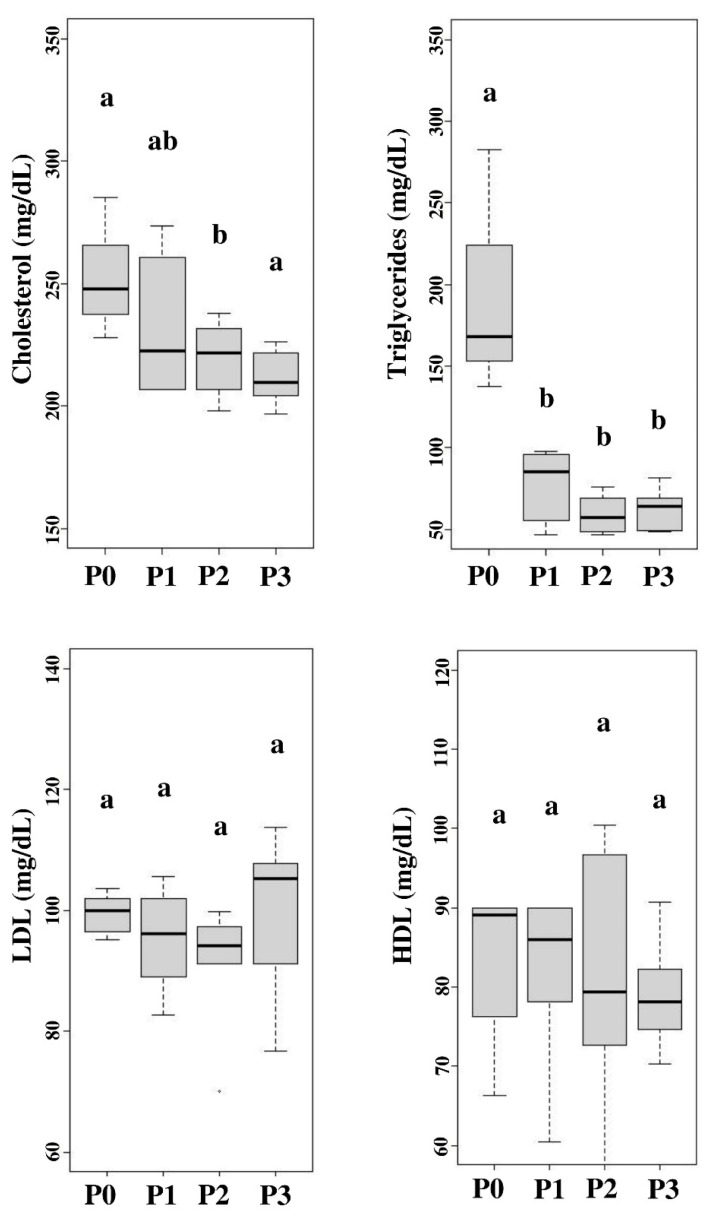
Blood lipid profile included blood cholesterol, triglyceride, low-density lipoprotein (LDL), and high-density lipoprotein (HDL) levels. P0, 100% basal diets+0% chicory; P1, 95% basal diets+5% chicory; P2, 90% basal diets+10% chicory; P3, 85% basal diets+15% chicory. ^a.b^ Means with different superscripts in a row differ significantly (p<0.05).

The blood lipid profile measured during the study included blood cholesterol, triglycerides, LDL, and HDL levels. Chicory supplementation showed lower (p<0.05) blood cholesterol and triglyceride levels than without chicory supplementation, while LDL and HDL in all treatments showed the same results (p>0.05). However, when viewed from the efficiency of its use, 15% supplementation was recommended among all treatments. Figure 3 shows the chicory supplementation on blood lipid profile including blood cholesterol, triglyceride, LDL, and HDL levels.

The low blood cholesterol level is thought to be due to the flavonoid content in chicory which can lower the duck blood cholesterol serum. This statement matches with Ouyang et al [4] who stated that flavonoids have many bio functions, one of which can reduce cholesterol levels by regulating HMG-CoA levels by increasing bile acids and increasing the rate of turnover of blood and liver cholesterol. HMG-CoA is crucial in cholesterol synthesis control, namely phosphorylation by inactivating enzymes to reduce cholesterol synthesis. Under conditions of starvation, cholesterol synthesis will be inhibited but cholesterol will increase if high sugar consumption and feed contain saturated fat.

However, hormone regulation is more complex. Insulin can induce hepatic HMG-CoA reductase synthesis, but cortisol and glucagon can inhibit and decrease enzyme activity. In addition, thyroid hormone can increase enzyme synthesis and help the transition of cholesterol to liver bile acids at the same time. Cholesterol synthesis freely forms very-low-density lipoprotein (VLDL) which is then excreted into the blood together with other lipids and apolipoproteins then utilized by other tissues and organs. Chicory also contains saponins which can lower the cholesterol content of meat and blood cholesterol. Saponins are glycosides found in many plants. Its characteristics of foam and emulsion can be used to lower cholesterol [8].

Saponins reduce blood plasma cholesterol levels by binding to bile acids so that the excretion of bile acids occurs in the feces of neutral sterols (coprostanol and cholesterol) [31]. This promotes a significant increase in the conversion of cholesterol to bile acids to sustain bile acid depots. As a consequence, LDL receptors from the liver will be expanded, leading to an increase in LDL absorption and a drop in blood plasma cholesterol levels [32]. Decreased triglyceride levels in the blood can be caused by the content of feed consumed such as carbohydrates and CF. The CF content can affect the level of lipid absorption so that in the end it will reduce blood triglyceride levels. Saponins can increase the production and secretion of bile, increase bile solid particles to be excreted, and accelerate fat metabolism to reduce blood triglycerides. Jiang et al [3] showed that the use of alfalfa as much as 3% can reduce triglycerides and VLDL. The chicory supplementation treatment showed low triglycerides due to the flavonoid content in the chicory plant. This is supported by the findings of Ouyang et al [4] who reported that bioflavonoids can lower cholesterol, triglycerides, and LDL. Flavonoids in chicory can increase lipoprotein lipase enzyme activity, thereby cause hydrolysis and VLDL. The lipoprotein transports triglycerides into fatty acids and glycerol. These fatty acids are subsequently taken by muscles and tissues, then are oxidized to generate energy. Adipose tissue then stores it as energy reserves [27]. The content of flavonoids can also inhibit fatty acid synthase (FAS). The inhibition of FAS lowers fatty acids formation and then declines the triglycerides synthesis.

Chicory supplementation had no significant (p>0.05) effect on LDL levels in the blood. However, it decreased numerically in the treatment given chicory supplementation. Jiang et al [3] stated that giving 9% alfalfa to ducks was able to reduce levels of fat, triglycerides and total cholesterol, LDL, and VLDL concentrations. High LDL levels are influenced by VLDL catabolism in the body. The VLDL levels are the chief of LDL formation. Increased VLDL in the blood will be followed by an increase in blood LDL levels [33]. Xu et al [34] mentioned that VLDL catabolism forms LDL, so LDL is rich in cholesterol. The LDL levels have a function as cholesterol transport in the blood to body tissues through endocytosis. High LDL levels indicate high cholesterol levels which are not good for health.

The LDL is a lipoprotein that is classified as bad fat because it binds to cholesterol and transports it to the tissues to target cells. Excessive LDL levels will precipitate and settle on artery walls and then HDL lipoprotein will pick up the scattered cholesterol to be carried back to the liver. The mechanism of chicory saponins lowering total cholesterol is in the formation of insoluble complex saponins that bind to cholesterol. Therefore, it can reduce cholesterol levels. The flavonoids in chicory can lower LDL levels in the blood. This is in line with the findings of Ouyang et al [4] who stated that flavonoids can reduce total cholesterol and serum LDL, but also can increase HDL in the blood in broilers. LDL is considered bad cholesterol that can cause atherosclerosis which is the main factor causing coronary heart disease. The mechanism of saponins lowering cholesterol is to inhibit the HMG-CoA reductase enzyme that performs cholesterol synthesis. Saponins also suppresses the acyl-CoA cholesterol O-acyltransferase 2 (ACAT_2_) enzyme which has an important function in atherogenic lipoproteins production. It also increases cholesterol excretion by increasing cholesterol 7-alpha-hydroxylase (CYP7A1) which plays a role in the breakdown of serum and liver cholesterol, as well as increases LDL receptor activity. Fiber consumption is known to significantly reduce LDL cholesterol levels [35].

Jiang et al [3] stated that giving 9% alfalfa to ducks was able to reduce HDL levels. Saponins in chicory can bind to endogenous cholesterol formed in bile and prevent cholesterol reabsorption. This is thought to be the reason saponins can lower LDL but do not affect HDL. In contrast to the findings of Ouyang et al [4], flavonoid content was not only lowering LDL levels but also increasing HDL levels in the blood. This study did not give significant results, but from a numerical point of view, the treatment using chicory as feed supplementation had a higher HDL value than the treatment without chicory. The antioxidant content of chicory is thought to increase liver Apo A1 mRNA which plays a role in initiating the synthesis of Apo A1 which is the main component of HDL. A decrease in HDL levels can be caused by an increase in triglyceride content. The HDL is a lipoprotein that regulates the cholesterol levels so that it does not build up in cells. The balance is maintained by the sterols removal from the membrane at a rate equal to the amount of cholesterol synthesized to the liver. Meanwhile, LDL plays a function in delivering cholesterol in body tissues since it is the primary transporter of cholesterol from the liver to body tissues. Thus, LDL levels in the blood are influenced by cholesterol concentrations.

### Score evaluation of chicory treatment

**Figure 4:**
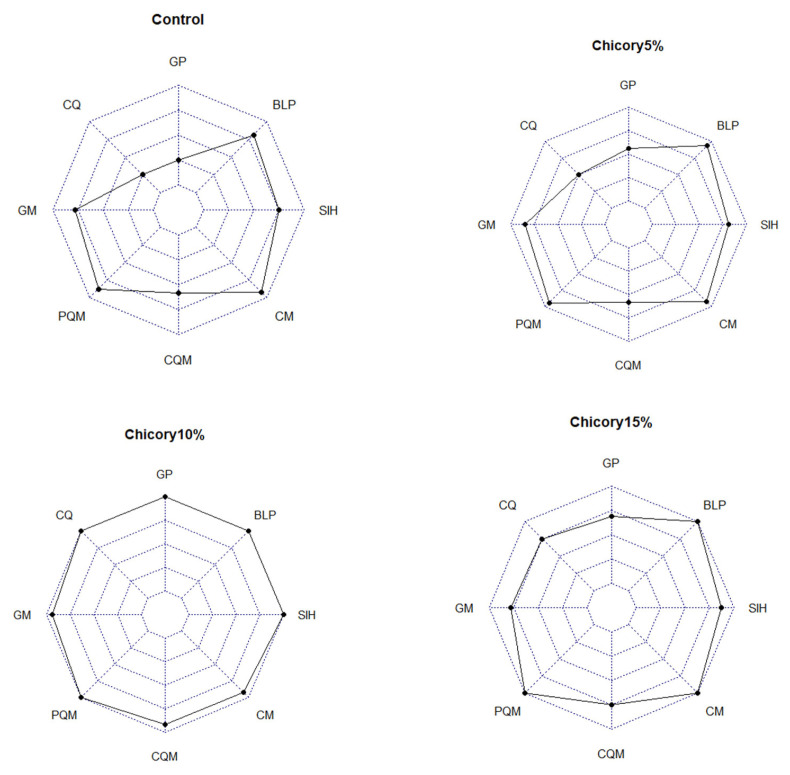
Score evaluation of chicory treatment. GP, growth performance; CQ, carcass quality; GM, gastrointestinal measurement; PQM, physical quality of meat; CQM, chemical quality of meat; CM, cholesterol of meat; SIH, small intestinal histomorphology; BLP, blood lipid profile.

The score evaluation in this study is shown in Table 6. Based on Table 6, it is known that chicory supplementation from highest to lowest was chicory 10%, 15%, 5%, and 0%. This means that chicory supplementation in this study showed optimal results but at a certain level decreased. These results are shown in Figure 4.

Growth performance was correlated with the quality of the carcass produced. Likewise, the nutrient content consumed will affect the nutrients that can be digested in the body. Ducks have a larger cecum than other poultry. This is because the duck's cecum is dominated by fiber-degrading microbes, namely cellulolytic and semi-cellulolytic. At a certain level, fibrous feed supplementation is very appropriate to be applied to ducks marked by positive changes. Under normal conditions, livestock will give a good response when given feed with a mixture of fiber which reduces the costs that must be incurred by farmers for feed.

## CONCLUSION

It can be concluded that chicory supplementation can improve the growth performance of hybrid duck, but at 15% level it causes a decrease. In this study, 10% chicory supplementation showed the best results characterized by higher growth performance, carcass quality, small intestinal histomorphology, and lower cholesterol levels of meat.

## Figures and Tables

**Table 1:** Chemical composition of feed ingredients (100% dry matter)

Ingredients	ME (MJ/kg)	CP (%)	CF (%)	EE (%)	Ca (%)	P (%)	Lys (%)	Met (%)	Tryp (%)
*Cichorium intybus*	2,620	15.53	14.23	2.39	0.29	0.01	1.51	0.19	0.00
Corn	3,321	8.90	2.20	4.00	0.02	0.23	0.29	0.18	0.08
Bran	2,887	12.00	5.20	10.7	0.04	1.27	0.50	0.19	0.10
Soybean meal	3,241	44.60	4.40	1.10	0.29	0.60	2.56	0.50	1.00
Fish flour	3,024	52.60	2.20	6.80	5.68	3.73	3.97	1.30	0.45
Wheat pollard	2,103	16.10	6.60	4.50	0.09	0.78	0.77	0.26	0.21
Minerals and vitamins premix	0	0.00	0.00	0.00	20.00	0.22	0.002	0.013	0.00

Analysis results from Forage and Pasture Laboratory, Faculty of Animal Science, Universitas Gadjah Mada.

ME, metabolizable energy; CP, crude protein; CF, crude fibre; EE, extract ether; Ca, calcium; P, phosphorus; Lys, lysine; Met, methionine; Tryp, tryptophane.

**Table 2:** Feed formulation based on nutrient composition

Ingredients (%)	Treatment^1)^			

	P0	P1	P2	P3
*Cichorium intybus*	0	5	10	15
Corn	33	33	33	33
Bran	19	19	19	19
Soybean meal	25	25	25	25
Fish flour	6	6	6	6
Wheat pollard	16	11	6	1
Minerals and vitamins premix	1	1	1	1
Total	100	100	100	100
Nutrient content (%DM)^2)^				
Metabolizable energy (MJ/kg)	2,972.63	2,998.52	3,024.40	3,050.28
Crude protein (%)	22.09	22.07	22.04	22.01
Crude fiber (%)	4.00	4.38	4.76	5.14
Extract ether (%)	10.82	9.10	6.97	4.84
Calcium (%)	0.64	0.65	0.66	0.67
Phosphorus (%)	0.81	0.78	0.74	0.70
Lysine (%)	1.19	1.23	1.27	1.30
Methionine (%)	0.34	0.34	0.33	0.33
Tryptophane (%)	0.34	0.34	0.33	0.32

1) P0, 100% basal diets+0% chicory; P1, 95% basal diets+5% chicory; P2, 90% basal diets+10% chicory; P3, 85% basal diets+15% chicory.

2) The chemical composition calculated theoretically from analyses provided by Forage and Pasture Laboratory, Faculty of Animal Science, Universitas Gadjah Mada.

**Table 3:** Average growth performance and carcass quality of hybrid ducks fed diets with chicory supplementation

Parameters	Treatment (mean±standard deviation)^1)^				p-value

	P0	P1	P2	P3	
Growth performance					
Feed intake (g/head)	128.33±2.58^c^	135.00±0.00^b^	140.00±4.47^a^	138.33±2.58^ab^	<0.001
BWG absolute (g/head)	20.52±0.31^d^	30.92±0.74^c^	37.31±1.02^a^	34.64±0.22^b^	<0.001
BWG relative (%)	3.96±0.42^c^	5.26±0.22^b^	5.69±0.33^a^	5.58±0.34^ab^	<0.001
FCR	6.92±0.46^a^	4.42±0.13^b^	3.63±0.14^c^	4.20±0.19^b^	<0.001
Carcass quality					
Live weight (g)	959.68±9.41^d^	1,371.79±24.27^c^	1,563.42±10.54^a^	1,421.41±37.93^b^	<0.001
Slaughter weight (g)	851.35±58.24^d^	1,238.45±54.48^c^	1,446.75±48.89^a^	1,338.08±55.15^b^	<0.001
Carcass weight (g)	403.14±64.95^c^	663.91±76.99^b^	806.93±52.72^a^	719.71±44.17^b^	<0.001
Carcass percentage (%)	41.97±6.45^b^	48.37±5.29^a^	51.62±3.43^a^	50.64±2.87^a^	0.009

BWG, body weight gain; FCR, feed conversion ratio.

1) P0, 100% basal diets+0% chicory; P1, 95% basal diets+5% chicory; P2, 90% basal diets+10% chicory; P3, 85% basal diets+15% chicory.

a–d Means with different superscripts in a row differed significantly (p<0.05).

**Table 4:** Measurement of weight and pH of digestive organs after chicory supplementation

Gastrointestinal parameters	Treatment of chicory (mean±standard deviation)^1)^				p-value

	P0	P1	P2	P3	
Weight (g)					
Gizzard	45.74±10.31^b^	67.53±7.28^a^	63.32±4.35^a^	64.5±3.79^a^	<0.001
Duodenum	5.36±0.72	6.1±0.96	5.54±1.40	5.96±0.82	0.558
Jejunum	14.06±2.21	15.62±2.29	15.89±1.70	16.45±1.25	0.193
Ileum	13.43±3.22	14.68±1.65	15.99±1.47	14.61±1.59	0.252
Cecum	3.56±0.35^c^	4.12±0.77^bc^	5.12±0.87^a^	4.39±0.40^ab^	0.003
Colon	2.57±0.35	2.94±0.35	3.41±0.82	3.07±0.46	0.084
Pancreas	3.09±0.60	3.9±0.65	3.75±0.58	3.93±0.64	0.095
Liver	26.04±2.26	28.73±4.40	27.04±2.59	27.14±2.48	0.510
pH					
Gizzard	5.83±0.41^a^	4.83±0.41^b^	5.67±0.52^a^	5.33±0.52^ab^	0.006
Duodenum	6.00±0.00	6.00±0.00	5.83±0.41	6.17±0.41	0.292
Jejunum	6.00±0.00	6.00±0.00	6.00±0.00	6.00±0.00	0.413
Ileum	6.00±0.00	6.00±0.00	6.00±0.00	6.00±0.00	0.413
Cecum	6.83±0.41	6.33±0.52	6.83±0.41	6.50±0.55	0.206
Colon	6.67±0.52^a^	6.17±0.41^ab^	5.83±0.41^b^	6.33±0.52^ab^	0.041
Pancreas	6.00±0.00	6.00±0.00	6.17±0.41	6.00±0.00	0.413
Liver	5.83±0.41	6.00±0.00	5.83±0.75	5.83±0.41	0.905

1) P0, 100% basal diets+0% chicory; P1, 95% basal diets+5% chicory; P2, 90% basal diets+10% chicory; P3, 85% basal diets+15% chicory.

a–c Means with different superscripts in a row differed significantly (p<0.05).

**Table 5:** Average physical, chemical, and cholesterol quality of hybrid duck meat

Meat quality	Treatment of chicory (mean±standard deviation)^1)^				p-value

	P0	P1	P2	P3	
Physical					
WHC (%)	42.85±1.81^b^	46.67±4.70^ab^	51.27±6.89^a^	50.19±4.20^a^	0.025
Cooking losses (%)	29.10±2.75	28.63±1.98	26.19±1.71	27.72±3.70	0.271
Tenderness (g/cm^2^)	2.35±0.74	2.06±0.53	2.18±0.58	2.13±0.49	0.848
pH	6.27±0.61	5.74±0.42	5.85±0.51	5.76±0.59	0.307
Chemical (%)					
Water content	73.23±0.47^b^	75.02±0.65^a^	75.53±1.64^a^	74.37±0.45^a^	0.002
dry matter	26.77±0.47^a^	24.98±0.65^b^	24.47±1.64^b^	25.63±0.45^b^	0.002
Ash	2.27±0.20 ^a^	2.14±0.26^a^	1.73±0.23^b^	2.05±0.26^a^	0.005
Organic matter	97.73±0.20^b^	97.86±0.26^b^	98.27±0.23^a^	97.95±0.26^b^	0.005
Extract ether	2.75±0.55^a^	1.08±0.06^b^	0.45±0.54^c^	1.14±0.49^b^	0.002
Crude protein	26.79±0.64^ab^	26.65±0.32^b^	27.43±0.47^a^	25.59±0.75^c^	<0.001
Cholesterol (mg/100 g)					
Breast	54.55±1.13^a^	55.37±0.81^a^	54.57±0.22^a^	53.56±0.54^b^	0.005
Rib	63.02±1.27	63.30±2.19	63.67±0.73	63.18±0.89	0.868
Liver	47.60±0.54	47.47±0.72	47.63±0.16	47.89±0.20	0.482

WHC, water holding capacity.

1) P0, 100% basal diets+0% chicory; P1, 95% basal diets+5% chicory; P2, 90% basal diets+10% chicory; P3, 85% basal diets+15% chicory.

a–c Means with different superscripts in a row differ significantly (p<0.05).

**Table 6:** Score evaluation of chicory treatment

Selection criteria	Relative weight	Treatment scoring^1)^				Relative weight×treatment scoring			
	
		P0	P1	P2	P3	P0	P1	P2	P3
Growth performance	0.125	25	56	100	69	3.13	7.03	12.50	8.59
Carcass quality	0.125	25	50	100	75	3.13	6.25	12.50	9.38
Gastrointestinal measurement	0.125	78	84	94	78	9.77	10.55	11.72	9.77
Physical quality of meat	0.125	88	94	100	100	10.94	11.72	12.50	12.50
Chemical quality of meat	0.125	58	58	92	75	7.29	7.29	11.46	9.38
Cholesterol of meat	0.125	92	92	92	100	11.46	11.46	11.46	12.50
Small intestinal histomorphology	0.125	75	81	100	88	9.38	10.16	12.50	10.94
The blood lipid profile	0.125	81	94	100	100	10.16	11.72	12.50	12.50
Optimum score						65.23	76.17	97.14	85.55

1) P0, 100% basal diets+0% chicory; P1, 95% basal diets+5% chicory; P2, 90% basal diets+10% chicory; P3, 85% basal diets+15% chicory.
